# The Screening Accuracy of a Visually Based Montreal Cognitive Assessment Tool for Older Adult Hearing Aid Users

**DOI:** 10.3389/fnagi.2021.706282

**Published:** 2021-08-12

**Authors:** Nattawan Utoomprurkporn, Joshua Stott, Sergi G. Costafreda, Courtney North, Mary Heatley, Doris Eva Bamiou

**Affiliations:** ^1^UCL Ear Institute, London, United Kingdom; ^2^Faculty of Medicine, Chulalongkorn University, Bangkok, Thailand; ^3^Division of Psychology and Language Science, Faculty of Brain Sciences, University College London, London, United Kingdom; ^4^Division of Psychiatry, Faculty of Brain Sciences, University College London, London, United Kingdom; ^5^NIHR Biomedical Research Centre Hearing and Deafness, London, United Kingdom

**Keywords:** montreal cognitive assessment (MoCA), cognitive screening, auditory cognitive neuroscience, hearing impairment, older adult

## Abstract

**Objectives:**

This research aims to validate a modified visually based Montreal Cognitive Assessment for hearing-aid users (MoCA-HA). This population should be the target of cognitive screening due to high risk of developing dementia.

**Design:**

Case-control study.

**Setting:**

The participants were recruited from referral hearing-aid center and memory clinic in central London, United Kingdom.

**Participant:**

75 hearing-aid users were recruited. Of these, thirty were cognitively intact controls with hearing impairment (NC-HI); thirty had mild cognitive impairment with hearing impairment (MCI-HI); fifteen had dementia with hearing impairment (D-HI).

**Measurements:**

The baseline characteristics and analysis of the MoCA-HA for the NC-HI were recorded. The MoCA-HA performance of the MCI-HI cohort and D-HI cohort were also studied.

**Results:**

The cutpoint of <26 yields 93.3% sensitivity with 80% specificity in distinguishing MCI-HI from NC-HI. The specificity increased to 95.6% in screening for all cognitive impairment (MCI-HI and D-HI) from NC-HI.

**Conclusion:**

The MoCA-HA has been validated with a cutpoint which is comparable to the traditional MoCA. This tool may help clinicians to early identify older adult hearing-aid users for appropriate cognitive evaluation.

## Introduction

One in every three adults over the age of 65 suffer from disabling hearing loss (World Health Organization (WHO), 2012; WHO, 2018). A recent meta-analysis showed that hearing loss is a significant modifiable risk factor for dementia, with a pooled relative risk of 1.94 (95% CI [1.38-2.73]) (Livingston et al., 2017). These findings indicate that older adults with hearing loss should be targeted for cognitive screening as a high-risk population for dementia.

Since current available screening tools for cognitive impairment require patients to follow orally presented instructions, normal hearing thresholds are implicitly assumed when conducting the test. However, mishearing or misinterpreting the test instructions and test items due to hearing loss can lead to underestimations of cognitive ability (Dupuis et al., 2015). Timely diagnosis of dementia is critical in promoting positive patients outcomes (Prince et al., 2011), and the development of sensitive, valid and reliable dementia screening tools designed for a hearing-impaired population is of paramount importance.

The Montreal cognitive assessment (MoCA) has excellent validity in identifying mild cognitive impairment (MCI) compared to other commonly used screening tools (Ciesielska et al., 2016; Kopecek et al., 2016). However, previously proposed modifications of the MoCA for its use in hearing-impaired populations tend to introduce new problems. For example, delivering the test with auditory amplification, has lead to the variability of results across cognitive sub-categories; and omitting hearing-dependent items, has decreased the tool’s sensitivity for detecting cognitive impairment (Saunders et al., 2018; Shen et al., 2019).

Another possible way of MoCA modification is to adapt commonly used dementia screening tools for a visual as opposed to an auditory presentation. Lin et al. have demonstrated the utility of a visually adapted version of the MoCA for the severely hearing-impaired (HI-MoCA) by examining the performance of cognitively intact individuals with cochlear-implants (Lin et al., 2017). However, cochlear implants candidates differ in several ways from the broader target population of older adults with age-related hearing loss, as the former have severe to profound hearing loss to meet cochlear implantation criteria, while the latter has all severities of hearing impairment.

This study aims to expand upon Lin et al.’s work by examining the performance of the adapted MoCA in older adults with no restriction of hearing loss severity. Moreover, it aims to test the ability of the visually adapted MoCA to distinguish individuals with a diagnosis of MCI and dementia from those without and establish an optimum cutpoint. The cutpoint may differ from the traditional MoCA due to the different test delivery modality and cognitive ability of the hearing-impaired cohort (Utoomprurkporn et al., 2020b).

## Materials and Methods

The project was approved by the UK National Health Services (NHS) Ethical Committee IRAS247176. The study was under the University College London Joint Research Office (JRO) sponsorship ID 18/0306. The study protocol was registered in clinicaltrial.gov with Identifier: NCT03648502.

### Participants

#### Older Adults With Hearing Impairment Who Had Normal Cognition (NC-HI) Cohort

A sample of 30 adults aged ≥65 were recruited via recruitment flyers and posters distributed in the hearing aid center at the Royal National Throat Nose Ear Hospital (RNTNEH), London, United Kingdom. The inclusion criteria were age ≥65 years with documented hearing loss (currently wearing hearing aids and/or audiogram with a better ear puretone average at 500 Hz, 1000 Hz, 2000 Hz and 4000 Hz of ≥ 30 dB HL) who are not on the cochlear implant waiting list. To ensure participants in the study had normal cognition, only those with a General Practitioner’s Assessment of Cognition (GPCOG) score of equal 9 or GPCOG score = 5–8 with informant score = 4–6 were recruited (Brodaty et al., 2004).

#### Older Adults With Hearing Impairment Who Were Diagnosed With Mild Cognitive Impairment (MCI-HI) and With Dementia (D-HI) Cohort

A sample of 30 adults diagnosed with mild cognitive impairment (MCI-HI) and 15 with dementia (D-HI) aged ≥65 were recruited via clinician referral and research registry in the memory clinics at Camden and Islington NHS Foundation Trust, London, United Kingdom. The RNTNEH, where the control NC-HI cohort was recruited, is also based within the Camden and Islington borough. The diagnosis of MCI and dementia cases were based on the ICD-10 criteria (WHO., 2016).

The diagnostic assessment was done within NHS Memory Services, which are specialist diagnostic services for the assessment of patients with suspected dementia referred by primary care doctors. Diagnostic assessments are conducted by medical practitioners, under the supervision of consultant old-age psychiatrists, and following ICD-10 criteria. The assessment consists of a clinical interview of patient and collateral obtained from relative or friend, review of medical and psychiatric history, assessment of functional needs inclusive of sensory impairment, review of psychiatric and physical health needs medication, any use of alcohol and drugs and their potential impact of cognition, assessment of mental state and cognitive testing. The main tool for cognitive testing is Addenbrooke’s Cognitive Examination v3, a validated clinical tool for the diagnosis of mild cognitive impairment and dementia. Additional neuropsychological testing is used if required and the diagnostic assessment includes a brain scan if clinically appropriate after initial assessment. The recruitments were done within 2 weeks of their last follow-up with the service to ensure the current status of the diagnosis of MCI and dementia.

The inclusion criteria were age ≥65 years with documented hearing loss (currently wearing hearing aids and/or audiogram with a better ear hearing average at 500 Hz, 1000 Hz, 2000 Hz and 4000 Hz of ≥30 dB HL) who are not in the cochlear implant waiting list.

The exclusion criteria for all groups were uncorrected visual impairment; physical disability(s) which might inhibit performance on the written/drawing elements of the tests as evaluated by the researchers, and congenital/childhood-onset hearing loss (<18 years old age) as reported by participants.

### Measures

#### Hearing Measurement

Audiograms were conducted for every participant according to the British Society of Audiology protocol (British Society of Audiology, 2018) during the same visit as the cognitive assessment. The hearing thresholds were recorded at 250 Hz, 500 Hz, 1000 Hz, 2000 Hz, 4000 Hz, and 8000 Hz for both right and left ear. For analysis purposes, the pure-tone audiogram outcome measure was the average of the thresholds (Pure-tone average: PTA) in 500 Hz, 1000 Hz, 2000 Hz, and 4000 Hz of the better hearing ear.

#### Cognition

A version of the MoCA adapted for people with hearing impairment/hearing aids users was used. The original MoCA has a total score of 30 with 7 subcategories which are Visuospatial/executive, Naming, Memory (word recall), Attention, Language, Abstract and Orientation.

The hearing-impaired MoCA (HI-MoCA) developed by Lin et al. (2017) was used in an initial Patient Public Involvement (PPI) group of older adults with hearing aids volunteers. As part of the PPI process, additional feedback from the healthcare providers including psychiatrists, clinical psychologists, audiologists, hearing aids center manager, otolaryngologists, audiovestibular medicine physicians (users), and from older adults with dual sensory impairments (visual and auditory) was also incorporated to the feedback from older adults with hearing impairment. We used the PPI information to adapt the MoCA version 8.3 into a computer-based tool by using only visual input to make it suitable for older adults with all severities of hearing loss. This version also included the Memory Index Score (MIS) sub-task which was not present in the HI-MoCA. The scoring sheet and administration instructions were downloaded from www.mocatest.org.

In the final version, the instructions were presented visually on the screen *via* the Microsoft PowerPoint program. The tool was also adapted according to guidance for the visually impaired population to ensure good visibility for the older adults with possible visual and hearing impairments. The duration of each slide timed was set according to the previously published paper by Lin et al. (2017).

The slides were presented to the participants by the administrator. The participants told the administrator when they were ready to move on to the next slide. The administrator guided the participants to read the instruction on the screen without further explanation by the administrator. The decision to have the administrator progressing the test to the next slide was suggested by the PPI volunteers, since they judged that some older people may not be comfortable when operating computer screens.

The participants responded to each slide verbally except when they were prompted to draw in the visuospatial/executive sub-tests. Their responses for this task were recorded in the original record form (MoCA 8.3) which can be downloaded from www.mocatest.org. There were some changes from the Lin et al. (2017) version. The decision to use the original response from recorded by the test administrator was made since the older volunteers were not comfortable with the self-written response form used by Lin et al. (2017). Moreover, volunteer PPI participants felt that writing down the word recall response would act as additional practice and therefore may represent additional help to remember beyond the standard MoCA instructions and overestimate memory status. The sentences recall task (part of a Language sub-category) was also affected by their writing ability of such compound sentences which took longer than a verbal response.

The final MoCA used was the MoCA version for hearing aids users (MoCA-HA) which incorporated all the changes suggested by the volunteer end-users and the health care professionals. The test was completed within 15 min. The MoCA-HA was used for all the participants recruited in this study.

## Analysis

The sample size was calculated for using Receiver Operating Curve (ROC) analysis using the EasyROC tool (Goksuluk et al., 2016) in distinguishing individuals with MCI from those without. The alpha was set at 0.05 and beta at 0.8. The estimated effect size [predicted area under the curve (AUC)] was set at 0.70. The effect size for the calculation was much less than the AUC for the original MoCA = 0.85 (Roalf et al., 2013). This was purposely done to ensure a conservative sample size estimate in case the hearing-impaired version of the MoCA is less accurate than the original MoCA.

The statistical analysis was done with IBM statistic SPSS program version 25. The baseline characteristics of the NC-HI, MCI-HI, and D-HI were compared with one-way ANOVA with Tukey-Kramer *post hoc* analysis. When the baseline characteristics shown significant difference, subgroup analysis with matched controls was done as a sensitivity analysis method to account for the differences. Matched controls analysis was done by repeating the sensitivity analysis after eliminating each unmatched control case until the baseline characteristics of interest were matched.

The Receiver Operating Characteristic [ROC] curve was computed to evaluate the overall effectiveness of a newly developed binary outcome diagnostic tool, MoCA-HA. The calculation was made against the ICD-10 gold standard of the diagnosis of MCI and dementia. The AUC of the plot, which determines the diagnostic property of this tool was also conducted (Bradley, 1997). The higher AUC (closer to 1.00) indicates a better diagnostic property of the tool.

The appropriate cutpoint for distinguishing between the NC-HI and MCI-HI was identified with the highest Youden index (*J*) value via formula *J* = (sensitivity + specificity)−1 (Youden, 1950; Greiner et al., 2000). The Youden index was previously found to better indicate the appropriate cutpoint to classify the cohorts than traditional visual inspection of the ROC curve (Perkins and Schisterman, 2006).

## Results

### Baseline Characteristics Data

There were significant differences in the mean age and years of education of the NC-HI, MCI-HI, and D-HI cohort, *F*(2,72) = 12.43, *p* < 0.005 and *F*(2,72) = 10.47, *p* < 0.005, respectively, as shown in Table 1. No significant difference was found in the better-ear PTA of the 3 groups [*F*(2,72) = 0.24, *p* = 0.79] as demonstrated in Table 1.

**TABLE 1:** Baseline characteristics of the 3 cohorts.

Baseline characteristics	NC-HI (N = 30)	MCI-HI (N = 30)	D-HI (N = 15)	F	P-value
Age	75.27 (SD = 5.88)	83.80 (SD = 6.42)	80.80 (SD = 8.53)	12.43	<0.005
Education years	16.07 (SD = 3.69)	13.27 (SD = 4.17)	10.53 (SD = 3.87)	10.47	<0.005
Better-ear pure-tone average (PTA)	48.87 (SD = 18.05)	47.75 (SD = 14.90)	45.33 (SD = 14.14)	0.24	0.79

The mean age of the NC-HI cohort was significantly lower than the MCI-HI cohort by 8.53 years (95% confidence interval; CI 12.66, 4.40) (*p* < 0.005) and the D-HI cohort by 5.53 years (95% CI 10.59, 0.47) (*p* = 0.03). There was no significant difference between the MCI-HI and the D-HI mean ages (*p* = 0.34).

The mean years of education of the NC-HI cohort was significantly higher than the MCI-HI cohort by 2.80 years (95% CI 0.37, 5.23) (*p* = 0.02) and the D-HI cohort by 5.53 years (95% CI 2.56,8.50) (*p* < 0.005). There was no significant difference between the MCI-HI and D-HI mean years of education (*p* = 0.08).

Due to the differences in the age and years of education, subgroup analysis with matched controls was done. As a result, only 9 NC-HI controls aged over 76 years old were included for the subgroup analysis which demonstrated no significant difference between the age and education years compared with the cognitively impaired group.

### Overall MoCA Performance for the Cohorts

**FIGURE 1:**
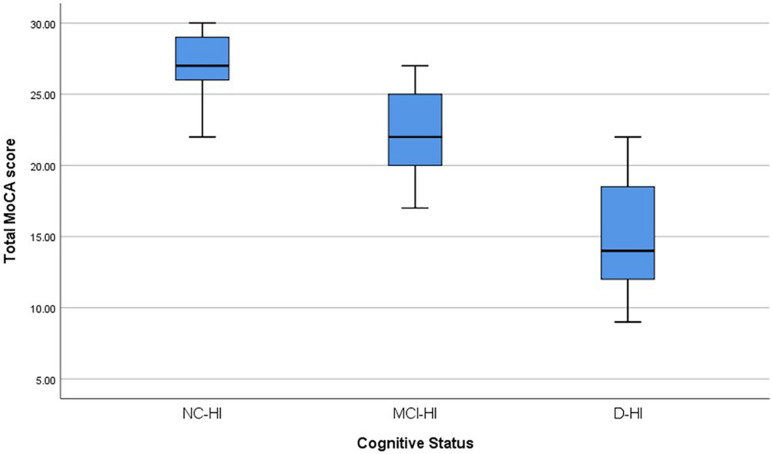
The Boxplots demonstrated the mean MoCA-HA score of the NC-HI, MCI-HI, and the D-HI cohorts.

**FIGURE 2:**
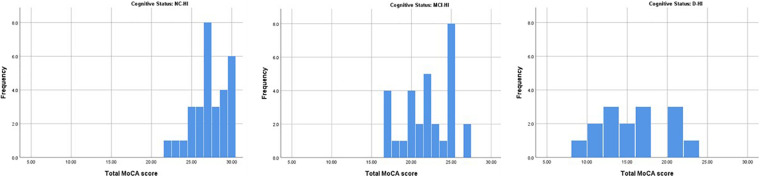
The histograms demonstrated the distribution of MoCA-HA score of the NC-HI, the MCI-HI and the D-HI cohort.

Overall; the total MoCA-HA mean score was 27.27(SD = 2.16) for the normal cognition (NC-HI) participants, and mean score = 22.03 (SD = 3.06) for the MCI-HI, and mean score = 15.20 (SD = 4.21) for the D-HI. The mean scores were significantly differenced among the three cohorts *F*(2,72) = 81.45 (*p* < 0.005) as demonstrated in Figure 1. The frequencies of MoCA-HA scores in each group were illustrated in Figure 2.

### Diagnostic Property of the MoCA-HA

#### For the Mild Cognitive Impairment Cohort

**FIGURE 3:**
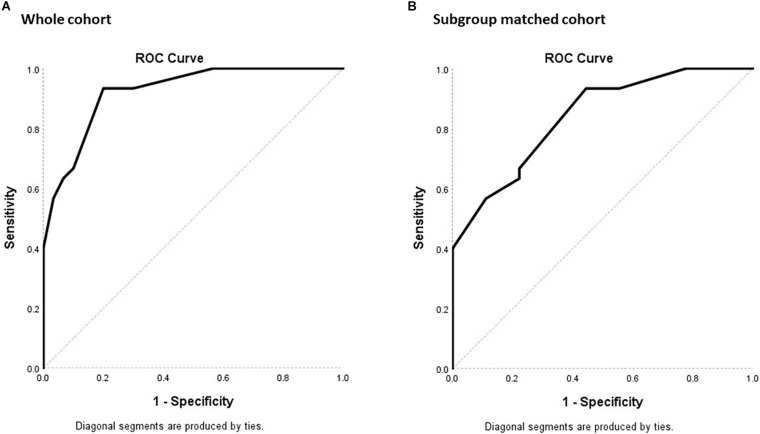
ROC plots of sensitivity against 1- specificity for the MoCA-HA tool of the NC-HI and the MCI-HI cohort to demonstrate the screening property of the tool. **(A)** The 30 NC-HI and 30 MCI-HI cohorts. **(B)** The age/education-matched 9 NC-HI and 30 MCI-HI cohorts. (The diagonal line demonstrates no significant diagnostic property for the dichotomous outcome with sensitivity = 50% and specificity = 50%).

For determining the diagnostic property of MoCA-HA in screening for MCI-HI, the AUC was calculated for the NC-HI and MCI-HI cohort (Figure 3). With the whole NC-HI cohort, the AUC was statistically significant at 0.92, standard error (SE) = 0.03 (95% CI 0.86, 0.99). The AUC with only aged and education-matched NC-HI controls (*N* = 9) was also statistically significant at 0.84 with SE = 0.07 (95% CI 0.70, 0.98).

With the whole NC-HI control cohort, the highest Youden index was 0.733, which resulted in the MoCA-HA cutpoint of 25.50 (sensitivity = 93.3%, specificity = 80%). With the matched NC-HI control cohort, the highest Youden index was 0.489, which resulted in the same MoCA-HA cutpoint score of 25.50 (sensitivity = 93.3%, specificity = 55.6%) in practice. The MoCA-HA only provides integer scores, therefore the overall score below 26 may be utilized as cutpoint.

#### For the Dementia Cohort (D-HI)

**FIGURE 4:**
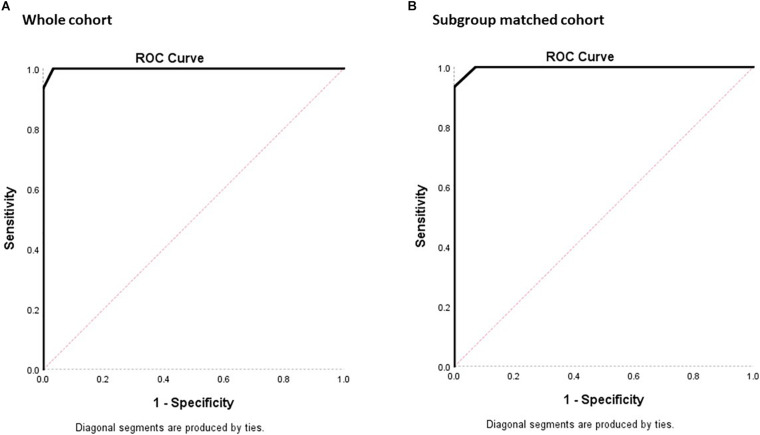
ROC plots of sensitivity against 1- specificity for the MoCA-HA tool of the NC-HI and the D-HI cohort to demonstrate the screening property of the tool. **(A)** The 30 NC-HI and 15 D-HI cohorts. **(B)** The matched 14 NC-HI and 15 D-HI cohorts. (The diagonal line demonstrates no significant diagnostic property for the dichotomous outcome with sensitivity = 50% and specificity = 50%).

For determining the diagnostic property of MoCA-HA in screening for D-HI, the AUC was calculated for the NC-HI and D-HI cohort (Figure 4). With the whole NC-HI cohort, the AUC was statistically significant at 0.999, SE = 0.002 (95% CI 0.994, 1.000). The AUC with aged-matched NC-HI controls (*N* = 14) was also statistically significant at 0.998, SE = 0.005 (95% CI 0.988, 1.000).

With the whole NC-HI control cohort, the highest Youden index was 0.967 which resulted in the MoCA-HA cutpoint of 22.5 (sensitivity = 100%, specificity = 96.7%).

With the 14 aged-matched NC-HI control cohort, the highest Youden index was 0.933 which resulted in the MoCA-HA cutpoint of 21.5 (sensitivity = 93.3%, specificity = 100%). The second highest Youden index was 0.929 which resulted in the MoCA-HA cut-point of 22.5 (sensitivity = 100%, specificity = 92.9%). In practice, the MoCA-HA only provides integer scores, therefore the overall score below 23 may be utilized as cutpoint to maximize the sensitivity of screening for dementia.

## Discussion

We have demonstrated the use of MoCA-HA (visually modified MoCA) for the hearing-impaired older adults. When implemented among adults aged ≥65 who used hearing aids, the MoCA-HA had an outstanding diagnostic property with AUC of >0.9. When considering only the matched control cohort of mean age >80 years, the MoCA-HA still has an excellent AUC of 0.84 (AUC 0.8–0.9).

The cutpoint of the MoCA-HA was found to be <26 which is similar to the traditional MoCA cutpoint in screening for the MCI-HI. This cutpoint also yield similarly high sensitivity of 93.3% as the traditional MoCA with a specificity of 80.0%. When considering all cognitive impairment stages (MCI-HI and D-HI), using a cutpoint of <26 could screen for potential cognitive impairment with even higher sensitivity up to 95.6% while maintaining the specificity of 80%.

### A Modified Version of MoCA for Hearing Loss/Hearing Aids Users (MoCA-HA)

It is important to have a version of the MoCA suitable for older adults with hearing impairment since previous research has shown that they may be at a higher risk of developing MCI or dementia (Livingston et al., 2017) and interpretation of the results of standard versions of the test are confounded by verbal presentation, with key dementia-relevant elements of registration, recall and attention, particularly affected (Al-Yawer et al., 2019).

Since cochlear implant candidates with prolonged and severe to profound hearing loss may have poorer speech production pre-operatively (Dawson et al., 1995), a written response for the MoCA may be more appropriate as used by Lin et al. (2017). However, for the majority of older adults who attend memory services or general practices, a written response may not be the best option to evaluate their cognition. Writing depends on additional fine motor skills in addition to the cognitive abilities that the MOCA measures, so that reduced performance assessed by written response may be due to impairment in these motor skills rather than on the target cognitive abilities. According to our PPI sessions, this added complexity may also cause confusion and stress for participants sessions. A verbal response was much more acceptable and comfortable among all older adults in the PPI interviews. Therefore, we decided to use the original scoring sheet of MoCA with the traditional verbal response from the subjects.

Another difference among cochlear implant candidates and other older adults with hearing impairment (hearing aids users) is that most of the cochlear implant candidates need to be physically fit enough to undergo surgery. Moreover, the candidates may tend to be younger since cochlear implant at a younger age was associated with a better outcome in older adults (Lin et al., 2012). In our PPI session, the participants preferred the test administrators to press the button for the next slide and to control the pacing of the task, since they were not comfortable with a computer screen. We implemented these changes in our protocol to enhance the participants testing experience and allow for standardized administration in future research and in clinical practice.

### Study Limitation

#### The Difference in Age and Education Years of the NC-HI From the MCI-HI and the D-HI Cohort

Since years of formal education has previously been found to be a risk factor for dementia (Livingston et al., 2017), lower education years among MCI-HI and D-HI were to be expected. The higher age of the MCI-HI and D-HI may be explained by our targeting of individuals who wore hearing aids. Despite an unusually high prevalence of hearing loss among patients in a memory clinic, individuals with cognitive impairment are known to be more likely to under-report their hearing difficulties and are therefore more likely to delay seeking medical intervention with hearing aid (Gold et al., 1996).

#### Role of Further Auditory Processing Disorder Evaluation

This study only evaluated the hearing ability of the cohorts by means of an audiogram. However, it is well established in the scientific literature that auditory processing disorder is also a possible diagnostic marker of cognitive dysfunction in older patients as well as peripheral type hearing loss. Further studies that apply an auditory processing test battery on these populations should be conducted in order to evaluate their hearing ability in more detail (Sardone et al., 2020; Utoomprurkporn et al., 2020a).

#### Generalizability of the Result

All recruitment and testing were at one site, which may limit generalizability. The findings of the study need to be validated at other sites and with larger samples. More sample with age/education years-matched controls could be beneficial in the implementation of this tool in a broader context.

### Clinical Implications and Further Research

Previous research has shown that performance on the original, verbally presented MoCA test performance is affected by hearing loss (Roalf et al., 2013), and while there is a visually presented version available (Lin et al., 2017), this requires a written response, which may be less practical and acceptable in a population of older users with hearing loss than the MoCA-HA, which require only verbal responses.

The MoCA-HA was well accepted by clinicians and patients. Our recently published work using the MoCA-HA has shown that the MoCA-HA results were not affected by the participants’ hearing levels (Utoomprurkporn et al., 2021). Using our modified version of MoCA will make it easier to disentangle the impact of hearing from cognitive impairment thus creating a more reliable tool for screening of cognitive impairment in this population for clinical and research purposes.

## Data Availability Statement

The raw data supporting the conclusions of this article will be made available by the authors, without undue reservation.

## Ethics Statement

The studies involving human participants were reviewed and approved by the UK National Health Services (NHS) Ethical Committee IRAS247176. The study was under the University College London Joint Research Office (JRO) sponsorship ID 18/0306. The patients/participants provided their written informed consent to participate in this study.

## Author Contributions

NU was the main author who conducted the research planning, recruitment/testing, and analysis under supervision of JS, SC, and DB. CN and MH conducted the recruitment and testing of D-HI participants. All authors contributed to the final manuscript.

## Conflict of Interest

The authors declare that the research was conducted in the absence of any commercial or financial relationships that could be construed as a potential conflict of interest.

## Publisher’s Note

All claims expressed in this article are solely those of the authors and do not necessarily represent those of their affiliated organizations, or those of the publisher, the editors and the reviewers. Any product that may be evaluated in this article, or claim that may be made by its manufacturer, is not guaranteed or endorsed by the publisher.
